# Crystal structure determination, Hirshfeld surface, crystal void, inter­molecular inter­action energy analyses, as well as DFT and energy framework calculations of 2-(4-oxo-4,5-di­hydro-1*H*-pyra­zolo[3,4-*d*]pyrimidin-1-yl)acetic acid

**DOI:** 10.1107/S2056989022008489

**Published:** 2022-08-31

**Authors:** Ezaddine Irrou, Younesse Ait Elmachkouri, Ali Oubella, Hassan Ouchtak, Samira Dalbouha, Joel T. Mague, Tuncer Hökelek, Lhoussaine El Ghayati, Nada Kheira Sebbar, Mohamed Labd Taha

**Affiliations:** a Laboratory of Organic and Physical Chemistry, Applied Bioorganic Chemistry Team, Faculty of Sciences, Ibn Zohr University, Agadir, Morocco; b Laboratory of Organic Synthesis and Molecular Physico-Chemistry, Department of Chemistry, Faculty of Sciences, Semlalia, BP 2390, Marrakech 40001, Morocco; c Laboratory of Organic Chemistry and Physical Chemistry, Research Team: Molecular Modeling, Materials and Environment, Department of Chemistry, Faculty of Sciences of Agadir, University Ibn Zohr, BP 8106 Agadir, Morocco; d Laboratory of Spectroscopy, Molecular Modeling, Materials, Nanomaterials, Water and Environment, CERNE2D, Faculty of Sciences, Mohammed V University in Rabat, Av. Ibn Battouta, BP 1014, Rabat, Morocco; e Department of Chemistry, Tulane University, New Orleans, LA 70118, USA; f Department of Physics, Hacettepe University, 06800 Beytepe, Ankara, Turkey; g Laboratory of Heterocyclic Organic Chemistry, Medicines Science Research Center, Pharmacochemistry Competence Center, Mohammed V University in Rabat, Faculty of Sciences, Av. Ibn Battouta, BP 1014, Rabat, Morocco; Vienna University of Technology, Austria

**Keywords:** crystal structure, hydrogen bond, C—H⋯π(ring) inter­action, pyrazolo­pyrimidine

## Abstract

In the title mol­ecule, C_7_H_6_N_4_O_3_, the bicyclic ring system is planar with the carb­oxy­methyl group inclined by 81.05 (5)° to this plane. In the crystal, corrugated layers parallel to (010) are generated by N—H⋯O, O—H⋯N and C—H⋯O hydrogen-bonding inter­actions. The layers are associated through C—H⋯π(ring) inter­actions. A Hirshfeld surface analysis indicates that the most important contributions to the crystal packing are from H⋯O/O⋯H (34.8%), H⋯N/N⋯H (19.3%) and H⋯H (18.1%) inter­actions. The volume of the crystal voids and the percentage of free space were calculated to be 176.30 Å^3^ and 10.94%, showing that there is no large cavity in the crystal packing. Computational methods revealed O—H⋯N, N—H⋯O and C—H⋯O hydrogen-bonding energies of 76.3, 55.2, 32.8 and 19.1 kJ mol^−1^, respectively. Evaluations of the electrostatic, dispersion and total energy frameworks indicate that the stabilization is dominated *via* dispersion energy contributions. Moreover, the optimized mol­ecular structure, using density functional theory (DFT) at the B3LYP/6–311G(d,p) level, was compared with the experimentally determined one. The HOMO–LUMO energy gap was determined and the mol­ecular electrostatic potential (MEP) surface was calculated at the B3LYP/6–31G level to predict sites for electrophilic and nucleophilic attacks.

## Abstract 2

The bicyclic ring system is planar with the carb­oxy­methyl group rotated out of this plane by 81.05 (5)°. In the crystal, corrugated layers are generated by N—H⋯O, O—H⋯N and C—H⋯O hydrogen-bonding inter­actions. The layers are associated through additional C—H⋯π(ring) inter­actions.

## 1. Chemical context

The chemistry of heterocyclic compounds still receives increasing inter­est because of the therapeutic importance of most heterocyclic compounds, especially those with nitro­gen-containing heterocycles, which are of great inter­est as potential bioactive mol­ecules (Taia *et al.*, 2020 ▸; Sebbar *et al.*, 2016 ▸; El Ghayati *et al.*, 2021 ▸; Dinakaran *et al.*, 2012 ▸; Lahmidi *et al.*, 2018 ▸; Hni *et al.*, 2019 ▸). In this regard, pyrazolo­[3,4-*d*]pyrimidines are an important family of heterocyclic compounds, and their derivatives possess various pharmacological properties (Bakavoli *et al.*, 2010 ▸; Severina *et al.*, 2016 ▸), including their applications as anti-microbial (Holla *et al.*, 2006 ▸; Bakavoli *et al.*, 2010 ▸), anti-tumor (Kandeel *et al.*, 2012 ▸), anti-inflammatory (El-Tombary, 2013 ▸), anti-oxidant (El-Mekabaty, 2015 ▸), anti-cancer (Gupta *et al.*, 2008 ▸; Maher *et al.*, 2019 ▸) and anti-convulsant (Severina *et al.*, 2016 ▸) agents. Pyrazolo­pyrimidines can also be used in the treatment of Alzheimers’ disease (Zhang *et al.*, 2018 ▸), and they have a powerful activity against herpes viruses (Gudmundsson *et al.*, 2009 ▸) and human leukaemia (HL-60) (Song *et al.*, 2011 ▸). 
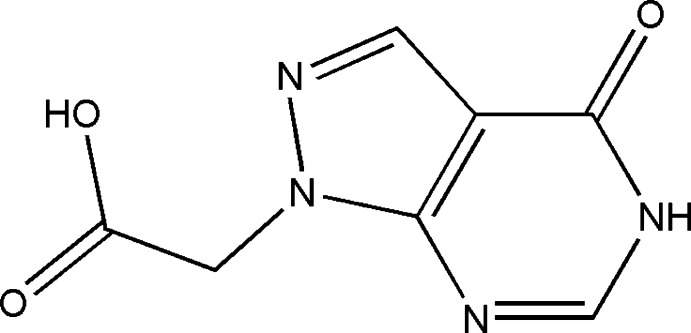


As a continuation of our research in this context, the title compound, (I), was synthesized by basic hydrolysis of the methyl ester of 2-(4-oxo-4, 5-di­hydro-1*H*-pyrazolo­[3,4-*d*]pyrimidin-1-yl)acetate. We report herein its synthesis, mol­ecular and crystal structures along with Hirshfeld surface analysis, crystal void and inter­molecular inter­action energies. Moreover, the optimized mol­ecular structure carried out at the B3LYP/6–311 G(d,p) level is compared with the experimentally determined structure, and energy framework as well as mol­ecular electrostatic potential (MEP) surface computations carried out at the B3LYP/6-31G level to predict the reactive sites for the electrophilic and nucleophilic attacks on (I) are presented.

## 2. Structural commentary

**Figure 1:**
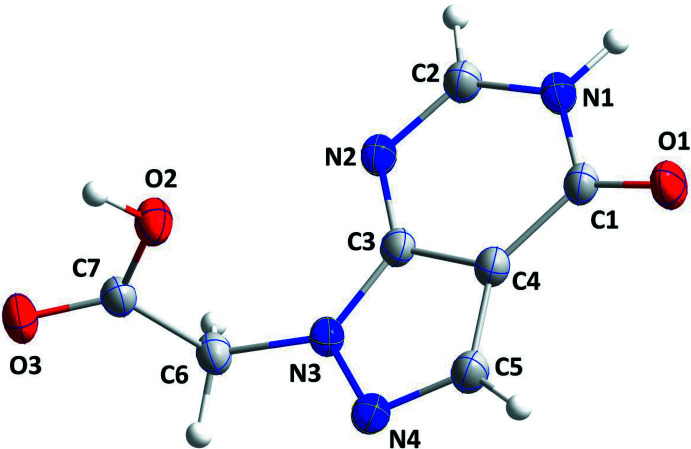
The title mol­ecule with displacement ellipsoids at the 30% probability level.

The pyrazolo­pyrimidine moiety is planar to within 0.0143 (10) Å (r.m.s deviation = 0.0082 Å) with the flap atom N1, which is part of the NH group, being the furthest from the mean plane. The plane of the carboxyl group is inclined to the above plane by 81.05 (5)°. There are no unusual bond lengths or inter­atomic angles in the mol­ecule (Fig. 1 ▸).

## 3. Supra­molecular features

**Figure 2:**
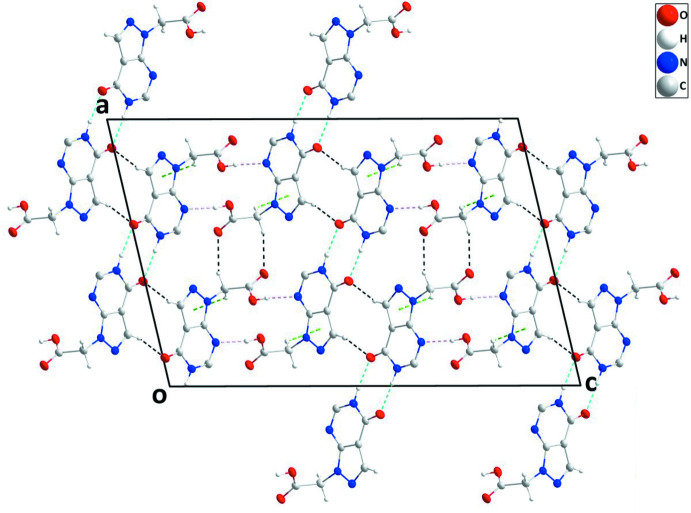
View of the crystal packing along the *b-*axis direction with N—H⋯O, O—H⋯N and C—H⋯O hydrogen bonds shown, respectively, by light-blue, pink and black dashed lines. C—H⋯π(ring) inter­actions are shown by green dashed lines.

**Figure 3:**
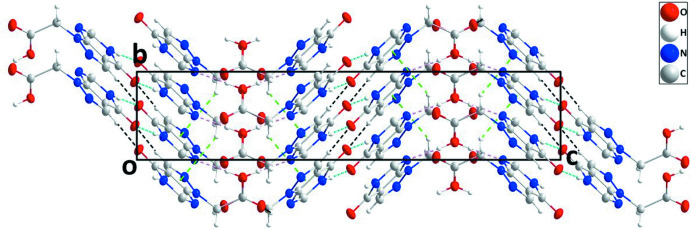
View of the crystal packing along the *a-*axis direction with N—H⋯O, O—H⋯N and C—H⋯O hydrogen bonds shown, respectively, by light-blue, pink and black dashed lines. C—H⋯π(ring) inter­actions are shown by green dashed lines.

In the crystal, inversion dimers are formed by N1—H1⋯O1 hydrogen bonds (Table 1 ▸) and are connected into sheets by complementary C5—H5⋯O1 hydrogen bonds (Table 1 ▸). Adjacent sheets are connected by O2—H2*A*⋯N2 and C6—C6*A*⋯O3 hydrogen bonds (Table 1 ▸) to form corrugated (010) layers with the component sheets alternately arranged parallel to (11 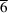 ) and ( 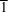 16) (Figs. 2 ▸ and 3 ▸). These corrugated layers are associated through C6—H6*B*⋯*Cg*1 inter­actions (Table 1 ▸ and Fig. 3 ▸).

## 4. Hirshfeld surface and crystal void analysis

**Figure 4:**
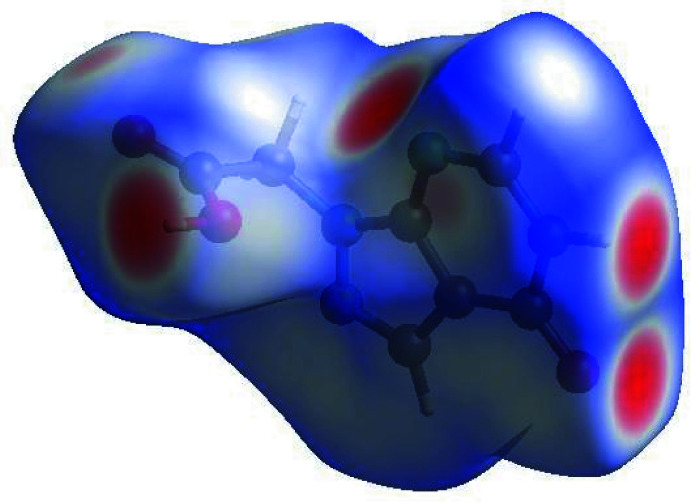
View of the three-dimensional Hirshfeld surface of the title compound, plotted over *d*_norm_ in the range −0.6986 to 1.3450 a.u.

**Figure 5:**
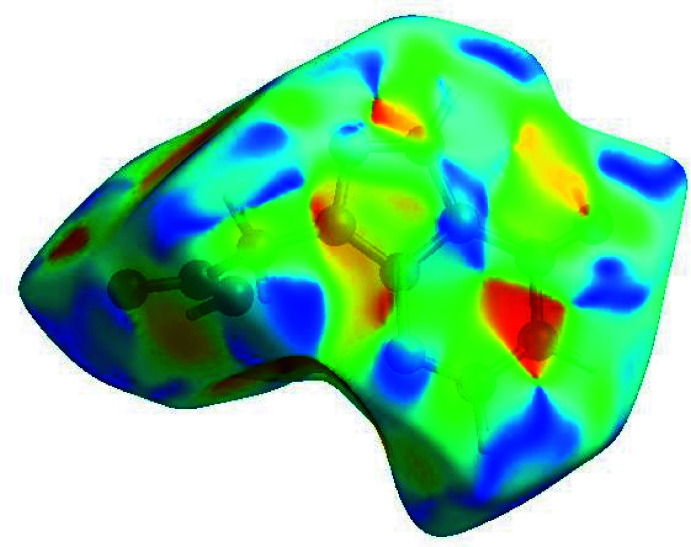
Hirshfeld surface of the title compound plotted over shape-index.

In order to visualize the inter­molecular inter­actions in the crystal of (I), a Hirshfeld surface (HS) analysis (Hirshfeld, 1977 ▸) was carried out by using *Crystal Explorer* (Spackman *et al.*, 2021 ▸). In the HS plotted over *d*_norm_ (Fig. 4 ▸), the white surface indicates contacts with distances equal to the sum of van der Waals radii, and the red and blue colours indicate distances shorter or longer than the sum of the van der Waals radii, respectively (Venkatesan *et al.*, 2016 ▸). The bright-red spots indicate sites of donor and/or acceptor inter­actions and they also appear as blue and red regions corresponding to positive (hydrogen bond donors) and negative (hydrogen bond acceptors) potentials on the HS mapped over electrostatic potential (Spackman *et al.*, 2008 ▸; Jayatilaka *et al.*, 2005 ▸), as shown in Fig. 5 ▸. The shape-index of the HS is a tool to visualize the presence of π–π stacking by the appearance of adjacent red and blue triangles. Fig. 5 ▸ clearly suggests that there are no significant π–π inter­actions in (I), as the above pattern is absent.

**Figure 6:**
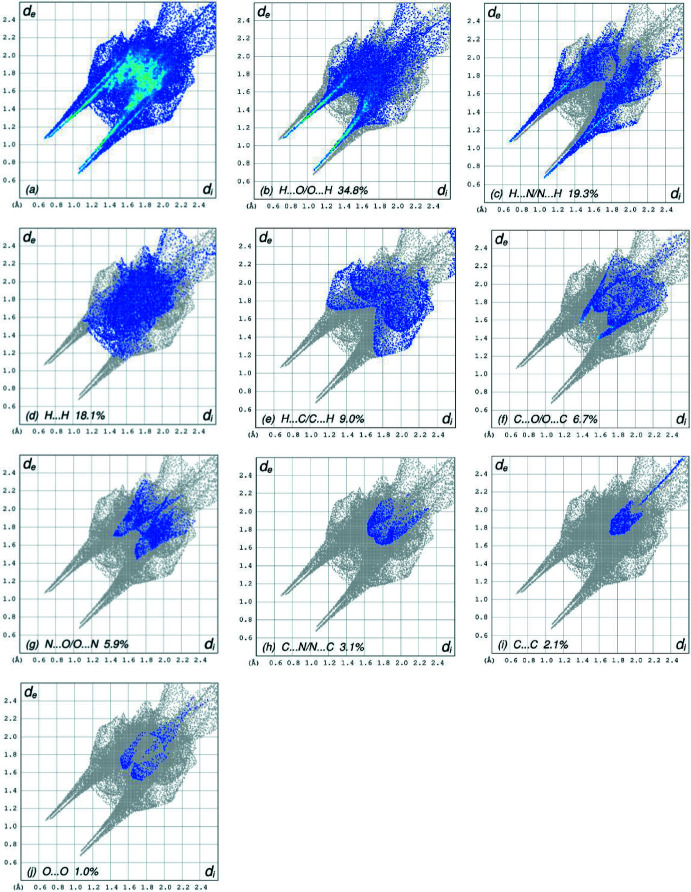
The full two-dimensional fingerprint plots for the title compound, showing (*a*) all inter­actions, and delineated into (*b*) H⋯O/O⋯H, (*c*) H⋯N/N⋯H, (*d*) H⋯H, (*e*) H⋯C/C⋯H, (*f*) C⋯O/O⋯C, (*g*) N⋯O/O⋯N, (*h*) C⋯N/N⋯C, (i) C⋯C and (*j*) O⋯O inter­actions. The *d*_i_ and *d*_e_ values are the closest inter­nal and external distances (in Å) from given points on the Hirshfeld surface contacts.

The overall two-dimensional fingerprint plot, Fig. 6 ▸ *a*, and those delineated into H⋯O/O⋯H, H⋯N/N⋯H, H⋯H, H⋯C/C⋯H, C⋯O/O⋯C, N⋯O/O⋯N, C⋯N/N⋯C, C⋯C and O⋯O contacts (McKinnon *et al.*, 2007 ▸), and their relative contributions to the Hirshfeld surface, are illustrated in Fig. 6 ▸ *b*–*j*. The most important inter­action is H⋯O/O⋯H, contributing 34.8% to the overall crystal packing, which is shown in Fig. 6 ▸ *b* where the pair of spikes have tips at *d*_e_ + *d*_i_ = 1.81 Å. The pair of spikes in the fingerprint plot delineated into H⋯N/N⋯H contacts, Fig. 6 ▸ *c*, with a 19.3% contribution to the HS has a symmetric distribution of points with the tips at *d*_e_ + *d*_i_ = 1.74 Å. The H⋯H contacts contribute with 18.1% to the HS and are shown in Fig. 6 ▸ *c* as widely scattered points of high density due to the large hydrogen content of the mol­ecule with the tip at *d*_e_ = *d*_i_ = 1.28 Å. The high contribution of these inter­actions suggest that van der Waals inter­actions play the major role in the crystal packing (Hathwar *et al.*, 2015 ▸). The presence of C—H⋯π inter­actions is shown by the pair of wings in the fingerprint plot delineated into H⋯C/C⋯H contacts with the tips at *d*_e_ + *d*_i_ = 2.93 Å (Fig. 6 ▸ *e*) with a 9.0% contribution to the HS. The C⋯O/O⋯C (Fig. 6 ▸ *f*), N⋯O/O⋯N (Fig. 6 ▸ *g*) and C⋯N/N⋯C (Fig. 6 ▸ *h*) contacts contribute with 6.7%, 5.9% and 3.1%, respectively, to the HS and the distributions of points appear with the tips at *d*_e_ + *d*_i_ = 2.95 Å, *d*_e_ + *d*_i_ = 3.15 Å and *d*_e_ + *d*_i_ = 3.38 Å. The C⋯C contacts, Fig. 6 ▸ *i*, with a 2.1% contribution to the HS, have a bullet-shaped distribution of points with the tip at *d*_e_ = *d*_i_ = 1.73 Å. Finally, the contribution of the O⋯O contacts (Fig. 6 ▸ *j*) to the HS is 1.0% with a low density of points.

**Figure 7:**
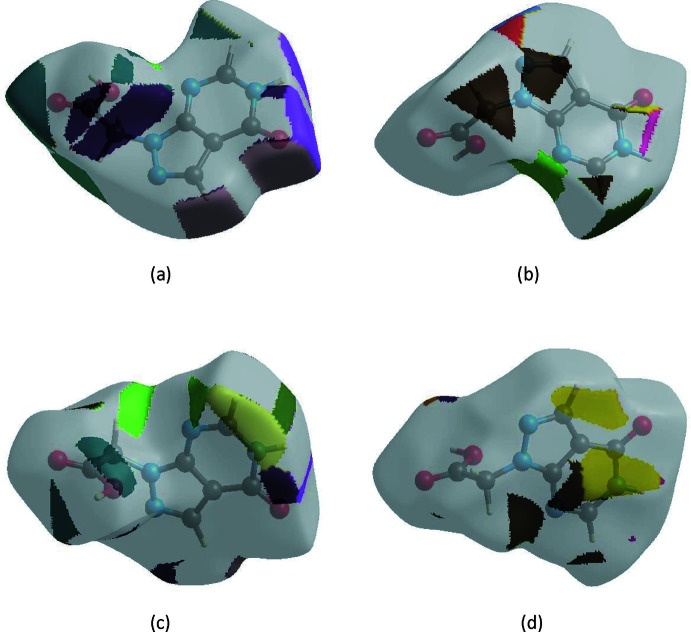
The Hirshfeld surface representations with the function *d*_norm_ plotted onto the surface for (*a*) H⋯O/O⋯H, (*b*) H⋯N/N⋯H, (*c*) H⋯H and (*d*) H⋯C/C⋯H inter­actions.

The Hirshfeld surface representations with the function *d*_norm_ plotted onto the surface are shown for the H⋯O/O⋯H, H⋯N/N⋯H, H⋯H and H⋯C/C⋯H inter­actions in Fig. 7 ▸ *a*–*d*, respectively.

**Figure 8:**
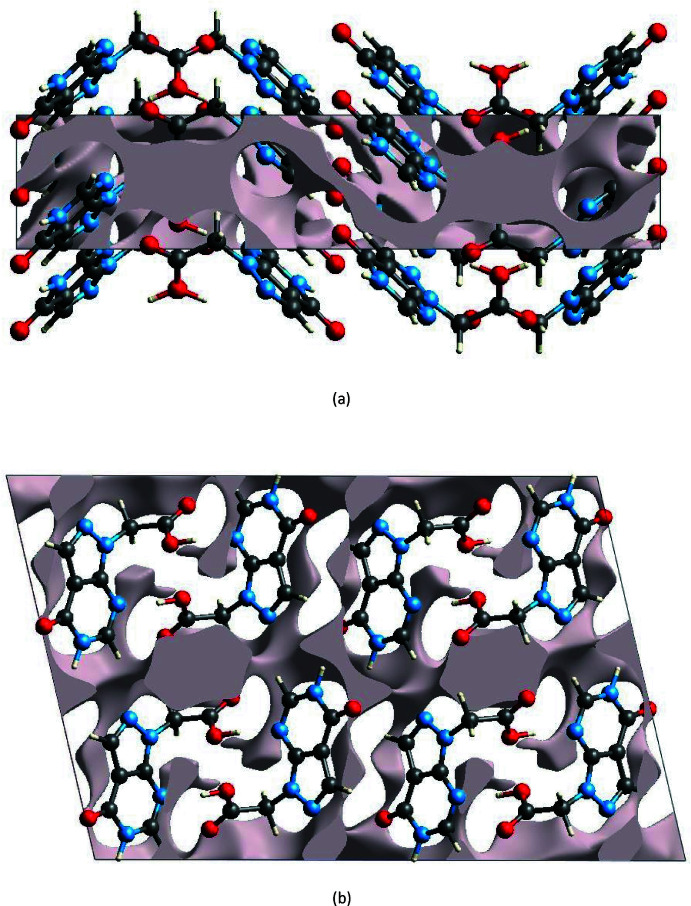
Graphical views of voids in the crystal packing of (I) (*a*) along the *a-*axis direction and (*b*) along the *b-*axis direction.

The strength of the crystal packing is important for determining the response to an applied mechanical force. If the crystal packing results in significant voids, then the mol­ecules are not tightly packed and a small amount of applied external mechanical force may easily break the crystal. To check the mechanical stability of the crystal, a void analysis was performed by adding up the electron densities of the spherically symmetric atoms contained in the asymmetric unit (Turner *et al.*, 2011 ▸). The void surface is defined as an isosurface of the procrystal electron density and is calculated for the whole unit cell where the void surface meets the boundary of the unit cell and capping faces are generated to create an enclosed volume. The volume of the crystal voids (Fig. 8 ▸ *a* and *b*) and the percentage of free space in the unit cell are calculated as 176.30 Å^3^ and 10.94%, respectively. Thus, the crystal packing appears compact and the mechanical stability should be substantial.

## 5. Inter­action energy and energy framework calculations

The inter­molecular inter­action energies were calculated using a CE–B3LYP/6–31G(d,p) energy model available in *Crystal Explorer* (Spackman *et al.*, 2021 ▸), where a cluster of mol­ecules is generated by applying crystallographic symmetry operations with respect to a selected central mol­ecule within the radius of 3.8 Å (Turner *et al.*, 2014 ▸). The total inter­molecular energy (*E*_tot_) is the sum of electrostatic (*E*_ele_), polarization (*E*_pol_), dispersion (*E*_dis_) and exchange–repulsion (*E*_rep_) energies (Turner *et al.*, 2015 ▸) with scale factors of 1.057, 0.740, 0.871 and 0.618, respectively (Mackenzie *et al.*, 2017 ▸). Hydrogen-bonding inter­action energies (in kJ mol^−1^) were calculated to be −73.0 (*E*_ele_), −16.4 (*E*_pol_), −27.3 (*E*_dis_), 93.6 (*E*_rep_) and −55.2 (*E*_tot_) for N1—H1⋯O1 hydrogen-bonding inter­actions, −103.5 (*E*_ele_), −22.1 (*E*_pol_), −13.2 (*E*_dis_), 98.8 (*E*_rep_) and −76.3 (*E*_tot_) for O2—H2*A*⋯N2 hydrogen-bonding inter­actions, −12.2 (*E*_ele_), −2.9 (*E*_pol_), −34.4 (*E*_dis_), 19.9 (*E*_rep_) and −32.8 (*E*_tot_) for C5—H5⋯O1 hydrogen-bonding inter­actions, and −19.5 (*E*_ele_), −3.2 (*E*_pol_), −12.3 (*E*_dis_), 23.8 (*E*_rep_) and −19.0 (*E*_tot_) for C6—H6*A*⋯O3 hydrogen-bonding inter­actions.

**Figure 9:**
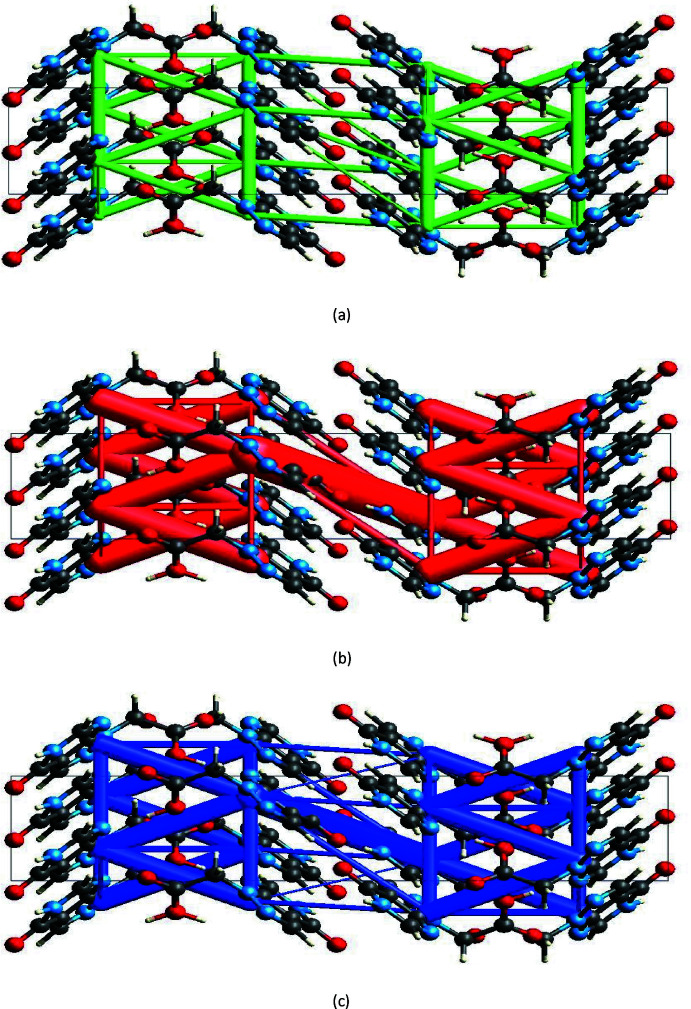
The energy framework for a cluster of mol­ecules of (I) viewed down the *a-*axis direction (the *b* axis is vertical and the *c* axis is horizontal) showing (*a*) electrostatic energy, (*b*) dispersion energy and (*c*) total energy diagrams. The cylindrical radius is proportional to the relative strength of the corresponding energies and they were adjusted to the same scale factor of 80 with cut-off value of 5 kJ mol^−1^ within 1 × 1 × 1 unit cells.

Energy frameworks combine the calculation of inter­molecular inter­action energies with a graphical representation of their magnitude (Turner *et al.*, 2015 ▸). Energies between mol­ecular pairs are represented as cylinders joining the centroids of pairs of mol­ecules with the cylinder radius proportional to the relative strength of the corresponding inter­action energy. Energy frameworks were constructed for *E*_ele_ (red cylinders), *E*_dis_ (green cylinders) and *E*_tot_ (blue cylinders) (Fig. 9 ▸ *a*, *b* and *c*). The results indicate that the stabilization is dominated *via* dispersion energy contributions in (I).

## 6. DFT and Mol­ecular electrostatic potential (MEP) calculations

Density functional theory (DFT) using the standard B3LYP functional (Becke, 1993 ▸) and Pople’s basis set 6–31G(d,p) implemented in *GAUSSIAN 09* (Frisch *et al.*, 2009 ▸) was used to optimize the mol­ecular structure of (I) in the gas phase. The minimum was confirmed by frequency calculations, and the resulting optimized parameters (bond lengths and angles) agreed satisfactorily with the experimental structural data (Table 2 ▸). As a result, energies and other physico-chemical properties obtained from the optimized structure could be safely used to describe those of (I). The corresponding HOMO and LUMO energies were then used to estimate some parameters of global chemical reactivity, such as electronegativity (χ), hardness (η), ionization potential (*I*), electrophilicity (ω) and softness (σ) (Table 3 ▸). In addition, the mol­ecular electrostatic potential (MEP) map, and dipole moment (μ) of (I) were similarly calculated.

Minor differences between theory and experiment are likely due to optimized values being obtained in the isolated gas phase, neglecting inter­actions in the solid phase. Briefly, the average O2—C7 and N4—C5 bond lengths calculated at the DFT/B3LYP/6-31G(d,p) level are 1.3767 and 1.3412 Å, respectively, which is slightly higher than the experimental values of 1.3148 (17) and 1.3182 (19) Å, respectively. Moreover, the torsion angles N4—N3—C3—N2 = −179.92° and C3—N2—C2—N1 = −0.53° agree well with the experimental ones of −178.88 (12) and −0.6 (2)°.

**Figure 10:**
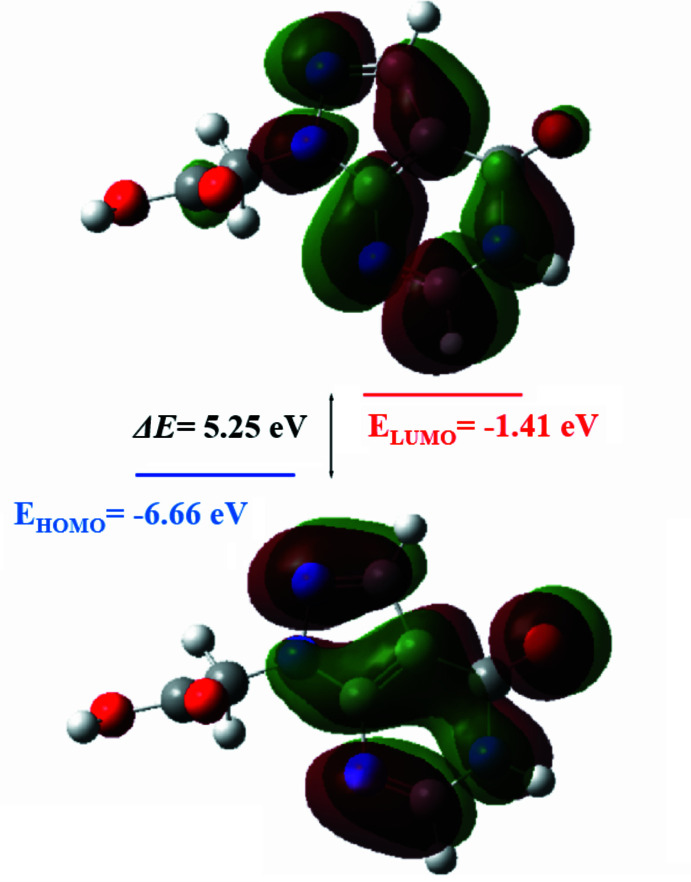
The optimized frontier molecular orbitals (HOMO and LUMO) with the energy band gap of (I).

The optimized frontier mol­ecular orbitals (HOMO and LUMO) are shown in Fig. 10 ▸. These orbitals play an important role in intra­molecular charge transfer (ICT). The topological characteristics of these levels are important for inter­preting kinetic stability and therefore potential chemical reactivity. The calculated energy band gap [Δ*E* = *E*_LUMO_ – *E*_HOMO_] of the mol­ecule is 5.25 eV which indicates a hard mol­ecule with low polarizability and low chemical and biological activities but high kinetic stability. The LUMO is mainly centered on the plane extending over the whole aromatic ring system of (I). The numerical reactivity descriptors, which are mainly based on HOMO–LUMO energies, are summarized in Table 3 ▸. The ionization potential (*I*) is defined as the amount of energy required to remove an electron from a mol­ecule. The high ionization energy indicates also high stability and hence chemical inertness. Electron affinity (*A*) is defined as the energy released when an electron is added to a neutral mol­ecule. Therefore a large value indicates the tendency of the mol­ecule to retain its electrons. A negative chemical potential (μ) reflects mol­ecular stability while hardness (η) characterizes the resistance of the cloud of mol­ecular electrons to deformation during small disturbances. The overall electrophilicity index (ω) of a mol­ecule is a measure of its stabilization energy following the addition of an external electronic charge or its resistance to exchange electron(s) with the system (Parr *et al.*, 1999 ▸). For (I), the ionization energy of 6.66 eV indicates high stability.

**Figure 11:**
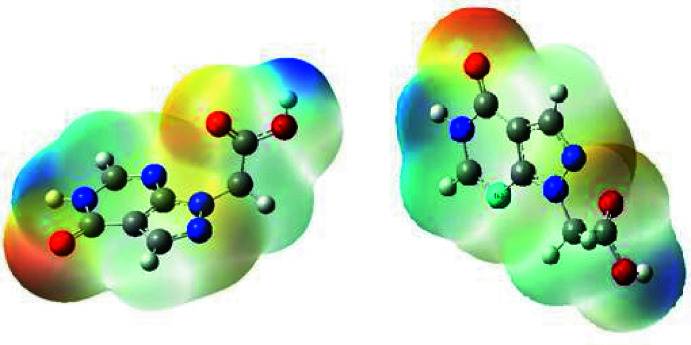
MEP surfaces of (I) mapped at the B3LYP/6–311G level.

**Figure 12:**
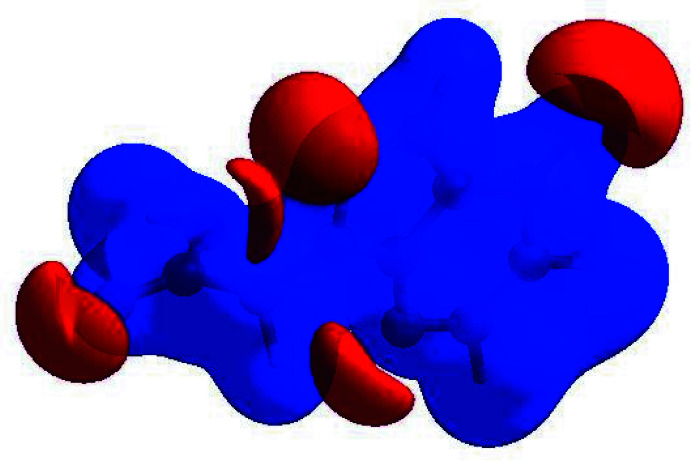
Contour surface of the electrostatic potential of (I).

The mol­ecular electrostatic potential (MEP) of the surface was calculated on the optimized B3LYP/6-31G (d,p) level using *Gaussview* software (Frisch *et al.*, 2009 ▸). The MEP surface (Fig. 11 ▸) gives information about the reactive sites. The total electron density on which the electrostatic potential surface has been mapped is shown in Fig. 12 ▸ where a visual representation of chemically active sites and the comparative reactivity of atoms is also shown. The red regions denote the most negative electrostatic potential, the blue regions represent most positive electrostatic potential, and green regions represent the region of zero potential. Fig. 12 ▸ confirms the existence of inter­molecular N—H⋯O, O—H⋯N and—H⋯O hydrogen-bonding inter­actions.

## 7. Database survey

**Figure 13:**
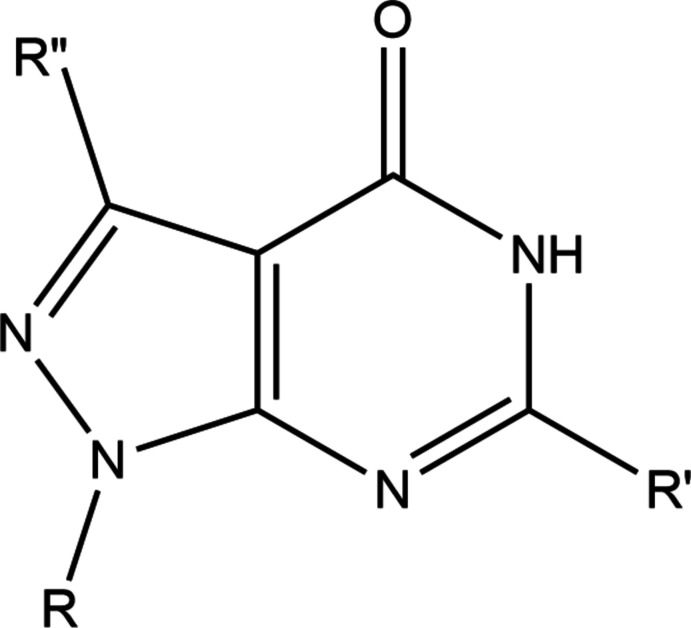
The mol­ecular moiety used for the database search procedure.

A search of the Cambridge Structural Database (CSD, updated March 2022; Groom *et al.*, 2016 ▸) with the search fragment **A** (*R* = C—CH, C—C—OH; *R*′ = *R*" = variable; Fig. 13 ▸) yielded 11 hits. These included structures with *R* = *t*-Bu, *R*" = H, *R*′ = Ph (RULHEN; Liu *et al.*, 2015 ▸), *p*-anis (QIBVIH; Tan *et al.*, 2007 ▸); *R*" = H, *R* = *i*-Pr, *R*′ = cyclo­butane­carboxamido (QIBVON; Tan *et al.*, 2007 ▸), *R* = *n*-Bu, *R*′ = benzamido (QIBWAA; Tan *et al.*, 2007 ▸), *R* = 3-phenyl­propyl, *R*′ = CH_3_S (IFICUV; Avasthi *et al.*, 2002 ▸), *R* = 2-chloro­ethyl, *R*′ = H (XAZRAT; Khazi *et al.*, 2012 ▸); *R* = 1-*β*-D-ribo­furanosyl, *R*′ = H, *R*" = OMe (FOVHIH; Anderson *et al.*, 1986 ▸), *R*′ = NH_2_, *R*" = H (YOMJIW; Ren *et al.*, 2019 ▸); *R* = 2-de­oxy-*β*-d-*erythro*-pento­furanosyl, *R*′ = NH_2_, *R*" = Br (HIPPAX; Seela *et al.*, 1999 ▸), *R*" = I (HIPPEB; Seela *et al.*, 1999 ▸); *R* = 2-de­oxy-2-fluoro-*β*-d-*arabino*-furanosyl, *R*′ = NH_2_, *R*" = Br (EJEJUY; He *et al.*, 2003 ▸). Like in (I), the pyrazolo­pyrimidine unit is essentially planar in these mol­ecules, but with the variety of substituents and the presence of different hydrogen-bonding inter­actions, the packings in the crystal are quite different.

## 8. Refinement

Crystal data, data collection and structure refinement details are summarized in Table 4 ▸. Hydrogen atoms attached to carbon were placed in idealized positions while those attached to nitro­gen and to oxygen were placed in locations derived from a difference-Fourier map and their parameters adjusted to give N—H = 0.91 and O—H = 0.87 Å. All H atoms were included as riding contributions with isotropic displacement parameters tied to those of the attached atoms.

## 9. Synthesis and crystallization

Ethyl 2-(4-oxo-4,5-di­hydro-1*H*-pyrazolo­[3,4-*d*]pyrimidin-1-yl)acetate (5 mmol) was dissolved in 10 ml of ethanol to which 10 ml of NaOH (aqueous, 10%_wt_) were added. The reaction mixture was stirred magnetically at room temperature for 4 h. After evaporation of ethanol and washing the aqueous phase with ethyl acetate, the mixture was acidified with an aqueous solution of HCl (3 *N*). The formed precipitate was filtered off and rinsed with ether. The crude product was recrystallized from ethanol to obtain colourless crystals of (I) in 72% yield.

## Supplementary Material

Crystal structure: contains datablock(s) I, global. DOI: 10.1107/S2056989022008489/wm5656sup1.cif

Supporting information file. DOI: 10.1107/S2056989022008489/wm5656Isup3.cdx

Supporting information file. DOI: 10.1107/S2056989022008489/wm5656Isup4.cml

Structure factors: contains datablock(s) I. DOI: 10.1107/S2056989022008489/wm5656Isup5.hkl

CCDC reference: 2203303

Additional supporting information:  crystallographic information; 3D view; checkCIF report

## Figures and Tables

**Table 1:** Hydrogen-bond geometry (Å, °) *Cg*1 is the centroid of the C3/C4/C5/N4/N3 ring.

*D*—H⋯*A*	*D*—H	H⋯*A*	*D*⋯*A*	*D*—H⋯*A*
N1—H1⋯O1^i^	0.91	1.90	2.8094 (16)	175
O2—H2*A*⋯N2^ii^	0.87	1.85	2.7120 (14)	173
C5—H5⋯O1^iii^	0.93	2.33	3.2305 (18)	164
C6—H6*A*⋯O3^iv^	0.97	2.36	3.2436 (18)	152
C6—H6*B*⋯*Cg*1^v^	0.97	2.98	3.7387 (15)	136

Symmetry codes: (i) 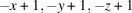 ; (ii) 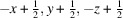 ; (iii) 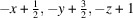 ; (iv) 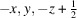 ; (v) 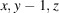 .

**Table 2:** Comparison of selected (X-ray and DFT) bond lengths and angles (Å, °)

	X-ray	B3LYP/6–311G(d,p)
O1=C1	1.2315 (17)	1.2450
O2—C7	1.3148 (17)	1.3767
O3=C7	1.2028 (17)	1.2273
N1—C2	1.3512 (18)	1.3749
N1—C1	1.3961 (18)	1.4047
N1—H1	0.9098	1.0128
N2=C2	1.3030 (18)	1.3131
N2—C3	1.3655 (17)	1.3742
N3—C3	1.3449 (17)	1.3605
N3—N4	1.3723 (16)	1.3955
N3—C6	1.4468 (17)	1.4417
C3=C4	1.3872 (19)	1.4048
N4=C5	1.3182 (19)	1.3412
C2—N1—C1	124.73 (11)	125.36
C2—N1—H1	121.4	120.93
C1—N1—H1	113.9	114.80
C2=N2—C3	112.91 (12)	113.46
C3—N3—N4	111.03 (11)	111.21
C3—N3—C6	128.49 (12)	128.12
N4—N3—C6	120.31 (11)	120.45
O1=C1—N1	120.51 (13)	120.02
O1=C1—C4	127.62 (13)	128.40
N2=C2—N1	124.94 (13)	124.12
O3=C7—O2	124.87 (13)	123.65
C2—N1—C1=O1	–176.82 (13)	–178.60
C3—N2—C2—N1	–0.6 (2)	–0.53
C1—N1—C2=N2	–1.4 (2)	–1.25
N4—N3—C3—N2	–178.88 (12)	–179.92

**Table 3:** Calculated energies

Total energy, *TE* (eV)	–19449.75
*E*_HOMO_ (eV)	–6.66
*E*_LUMO_ (eV)	–1.41
Gap, *ΔE* (eV)	5.25
Dipole moment, *μ* (Debye)	4.49
Ionization potential, *I* (eV)	6.66
Electron affinity, *A*	1.42
Electronegativity, *χ*	4.04
Hardness, *η*	2.62
Electrophilicity index, *ω*	3.11
Softness, *σ*	0.38
Fraction of electron transferred, *ΔN*	0.56

**Table 4:** Experimental details

Crystal data	
Chemical formula	C_7_H_6_N_4_O_3_
*M*_r_	194.16
Crystal system, space group	Monoclinic, *C*2/*c*
Temperature (K)	296
*a*, *b*, *c* (Å)	15.3747 (4), 4.6699 (1), 23.0423 (5)
β (°)	103.122 (1)
*V* (Å^3^)	1611.20 (6)
*Z*	8
Radiation type	Cu *K*α
μ (mm^−1^)	1.11
Crystal size (mm)	0.24 × 0.18 × 0.11

Data collection	
Diffractometer	Bruker D8 VENTURE PHOTON 100 CMOS
Absorption correction	Multi-scan (*SADABS*; Krause *et al.*, 2015 ▸)
*T*_min_, *T*_max_	0.81, 0.89
No. of measured, independent and observed [*I* > 2σ(*I*)] reflections	5850, 1598, 1503
*R*_int_	0.026
(sin θ/λ)_max_ (Å^−1^)	0.625

Refinement	
*R*[*F* ^2^ > 2σ(*F* ^2^)], *wR*(*F* ^2^), *S*	0.048, 0.114, 1.16
No. of reflections	1598
No. of parameters	128
H-atom treatment	H-atom parameters constrained
Δρ_max_, Δρ_min_ (e Å^−3^)	0.25, −0.33

Computer programs: *APEX3* and *SAINT* (Bruker, 2016 ▸), *SHELXT* (Sheldrick, 2015*a* ▸), *SHELXL* (Sheldrick, 2015*b* ▸), *DIAMOND* (Brandenburg & Putz, 2012 ▸) and *publCIF* (Westrip, 2010 ▸).

## supplementary crystallographic information

### Crystal data

**Table:**

C_7_H_6_N_4_O_3_	*F*(000) = 800
*M**_r_* = 194.16	*D*_x_ = 1.601 Mg m^−^^3^
Monoclinic, *C*2/*c*	Cu *K*α radiation, λ = 1.54178 Å
*a* = 15.3747 (4) Å	Cell parameters from 5261 reflections
*b* = 4.6699 (1) Å	θ = 3.9–74.4°
*c* = 23.0423 (5) Å	µ = 1.11 mm^−^^1^
β = 103.122 (1)°	*T* = 296 K
*V* = 1611.20 (6) Å^3^	Block, colourless
*Z* = 8	0.24 × 0.18 × 0.11 mm

### Data collection

**Table:**

Bruker D8 VENTURE PHOTON 100 CMOS diffractometer	1598 independent reflections
Radiation source: INCOATEC IµS micro–focus source	1503 reflections with *I* > 2σ(*I*)
Mirror monochromator	*R*_int_ = 0.026
Detector resolution: 10.4167 pixels mm^-1^	θ_max_ = 74.4°, θ_min_ = 3.9°
ω scans	*h* = −16→18
Absorption correction: multi-scan (*SADABS*; Krause *et al.*, 2015)	*k* = −5→5
*T*_min_ = 0.81, *T*_max_ = 0.89	*l* = −28→28
5850 measured reflections	

### Refinement

**Table:**

Refinement on *F*^2^	Secondary atom site location: difference Fourier map
Least-squares matrix: full	Hydrogen site location: mixed
*R*[*F*^2^ > 2σ(*F*^2^)] = 0.048	H-atom parameters constrained
*wR*(*F*^2^) = 0.114	*w* = 1/[σ^2^(*F*_o_^2^) + (0.063*P*)^2^ + 0.6265*P*] where *P* = (*F*_o_^2^ + 2*F*_c_^2^)/3
*S* = 1.16	(Δ/σ)_max_ < 0.001
1598 reflections	Δρ_max_ = 0.25 e Å^−^^3^
128 parameters	Δρ_min_ = −0.33 e Å^−^^3^
0 restraints	Extinction correction: *SHELXL 2018/3* (Sheldrick, 2015*b*), Fc^*^=kFc[1+0.001xFc^2^λ^3^/sin(2θ)]^-1/4^
Primary atom site location: dual	Extinction coefficient: 0.0304 (14)

### Special details

**Table:**

Geometry. All esds (except the esd in the dihedral angle between two l.s. planes) are estimated using the full covariance matrix. The cell esds are taken into account individually in the estimation of esds in distances, angles and torsion angles; correlations between esds in cell parameters are only used when they are defined by crystal symmetry. An approximate (isotropic) treatment of cell esds is used for estimating esds involving l.s. planes.
Refinement. Refinement of F^2^ against ALL reflections. The weighted R-factor wR and goodness of fit S are based on F^2^, conventional R-factors R are based on F, with F set to zero for negative F^2^. The threshold expression of F^2^ > 2sigma(F^2^) is used only for calculating R-factors(gt) etc. and is not relevant to the choice of reflections for refinement. R-factors based on F^2^ are statistically about twice as large as those based on F, and R- factors based on ALL data will be even larger. H-atoms attached to carbon were placed in calculated positions (C—H = 0.95 - 0.99 Å) while those attached to nitrogen and to oxygen were placed in locations derived from a difference map and their parameters adjusted to give N—H = 0.91 and O—H = 0.87 Å. All were included as riding contributions with isotropic displacement parameters 1.2 - 1.5 times those of the attached atoms.

### Fractional atomic coordinates and isotropic or equivalent isotropic displacement parameters (Å^2^)

**Table:**

	*x*	*y*	*z*	*U*_iso_*/*U*_eq_	
O1	0.39351 (7)	0.6126 (2)	0.49522 (5)	0.0517 (4)	
H1	0.491406	0.298878	0.457379	0.078*	
O2	0.17845 (7)	0.3011 (2)	0.24916 (4)	0.0474 (3)	
H2A	0.169736	0.370596	0.213269	0.071*	
O3	0.07906 (7)	−0.0274 (3)	0.20612 (5)	0.0586 (4)	
N1	0.43440 (7)	0.2658 (3)	0.43684 (5)	0.0417 (3)	
N2	0.33583 (7)	0.0013 (3)	0.36309 (5)	0.0397 (3)	
N3	0.18190 (7)	0.1198 (3)	0.35967 (5)	0.0375 (3)	
C1	0.37053 (9)	0.4290 (3)	0.45639 (6)	0.0389 (4)	
C2	0.41582 (9)	0.0694 (3)	0.39270 (6)	0.0421 (4)	
H2	0.463751	−0.024383	0.382697	0.051*	
C3	0.27031 (9)	0.1489 (3)	0.38153 (5)	0.0353 (3)	
C4	0.28199 (9)	0.3561 (3)	0.42577 (5)	0.0376 (3)	
N4	0.13470 (8)	0.3040 (3)	0.38734 (5)	0.0435 (3)	
C5	0.19481 (10)	0.4438 (3)	0.42717 (6)	0.0438 (4)	
H5	0.181431	0.582221	0.452827	0.053*	
C6	0.13600 (9)	−0.0569 (3)	0.31065 (6)	0.0410 (4)	
H6A	0.076673	−0.099206	0.316240	0.049*	
H6B	0.167753	−0.237123	0.311870	0.049*	
C7	0.12731 (8)	0.0757 (3)	0.24955 (6)	0.0387 (4)	

### Atomic displacement parameters (Å^2^)

**Table:**

	*U*^11^	*U*^22^	*U*^33^	*U*^12^	*U*^13^	*U*^23^
O1	0.0478 (6)	0.0593 (7)	0.0440 (6)	−0.0021 (5)	0.0021 (4)	−0.0198 (5)
O2	0.0562 (6)	0.0484 (6)	0.0337 (5)	−0.0094 (5)	0.0024 (4)	0.0066 (4)
O3	0.0537 (7)	0.0787 (8)	0.0370 (6)	−0.0145 (6)	−0.0030 (5)	−0.0075 (5)
N1	0.0377 (6)	0.0487 (7)	0.0362 (6)	−0.0022 (5)	0.0031 (5)	−0.0063 (5)
N2	0.0409 (6)	0.0435 (6)	0.0333 (6)	−0.0011 (5)	0.0057 (5)	−0.0058 (5)
N3	0.0373 (6)	0.0434 (6)	0.0300 (6)	−0.0026 (5)	0.0040 (4)	−0.0002 (4)
C1	0.0435 (7)	0.0425 (7)	0.0293 (6)	−0.0021 (6)	0.0050 (5)	−0.0036 (5)
C2	0.0418 (7)	0.0470 (8)	0.0366 (7)	0.0006 (6)	0.0069 (6)	−0.0051 (6)
C3	0.0380 (7)	0.0392 (7)	0.0278 (6)	−0.0021 (5)	0.0056 (5)	0.0016 (5)
C4	0.0405 (7)	0.0425 (7)	0.0290 (6)	−0.0013 (5)	0.0062 (5)	−0.0019 (5)
N4	0.0415 (6)	0.0525 (7)	0.0364 (6)	0.0021 (5)	0.0087 (5)	−0.0001 (5)
C5	0.0452 (8)	0.0510 (8)	0.0348 (7)	0.0026 (6)	0.0081 (6)	−0.0050 (6)
C6	0.0418 (7)	0.0433 (8)	0.0348 (7)	−0.0092 (6)	0.0018 (5)	−0.0003 (5)
C7	0.0354 (7)	0.0446 (8)	0.0336 (7)	0.0004 (5)	0.0024 (5)	−0.0018 (5)

### Geometric parameters (Å, º)

**Table:**

O1—C1	1.2315 (17)	N3—C6	1.4468 (17)
O2—C7	1.3148 (17)	C1—C4	1.4251 (18)
O2—H2A	0.8701	C2—H2	0.9300
O3—C7	1.2028 (17)	C3—C4	1.3872 (19)
N1—C2	1.3512 (18)	C4—C5	1.4090 (19)
N1—C1	1.3961 (18)	N4—C5	1.3182 (19)
N1—H1	0.9098	C5—H5	0.9300
N2—C2	1.3030 (18)	C6—C7	1.5160 (18)
N2—C3	1.3655 (17)	C6—H6A	0.9700
N3—C3	1.3449 (17)	C6—H6B	0.9700
N3—N4	1.3723 (16)		
			
C7—O2—H2A	110.0	C3—C4—C5	104.74 (12)
C2—N1—C1	124.73 (11)	C3—C4—C1	118.71 (12)
C2—N1—H1	121.4	C5—C4—C1	136.54 (13)
C1—N1—H1	113.9	C5—N4—N3	105.83 (11)
C2—N2—C3	112.91 (12)	N4—C5—C4	111.12 (13)
C3—N3—N4	111.03 (11)	N4—C5—H5	124.4
C3—N3—C6	128.49 (12)	C4—C5—H5	124.4
N4—N3—C6	120.31 (11)	N3—C6—C7	114.60 (11)
O1—C1—N1	120.51 (13)	N3—C6—H6A	108.6
O1—C1—C4	127.62 (13)	C7—C6—H6A	108.6
N1—C1—C4	111.86 (11)	N3—C6—H6B	108.6
N2—C2—N1	124.94 (13)	C7—C6—H6B	108.6
N2—C2—H2	117.5	H6A—C6—H6B	107.6
N1—C2—H2	117.5	O3—C7—O2	124.87 (13)
N3—C3—N2	125.93 (12)	O3—C7—C6	121.10 (13)
N3—C3—C4	107.27 (12)	O2—C7—C6	113.99 (11)
N2—C3—C4	126.80 (12)		
			
C2—N1—C1—O1	−176.82 (13)	O1—C1—C4—C3	177.49 (14)
C2—N1—C1—C4	2.4 (2)	N1—C1—C4—C3	−1.64 (18)
C3—N2—C2—N1	−0.6 (2)	O1—C1—C4—C5	−1.6 (3)
C1—N1—C2—N2	−1.4 (2)	N1—C1—C4—C5	179.24 (15)
N4—N3—C3—N2	−178.88 (12)	C3—N3—N4—C5	−0.97 (15)
C6—N3—C3—N2	−3.7 (2)	C6—N3—N4—C5	−176.57 (12)
N4—N3—C3—C4	0.92 (15)	N3—N4—C5—C4	0.64 (16)
C6—N3—C3—C4	176.06 (12)	C3—C4—C5—N4	−0.10 (16)
C2—N2—C3—N3	−179.00 (13)	C1—C4—C5—N4	179.09 (15)
C2—N2—C3—C4	1.2 (2)	C3—N3—C6—C7	−83.22 (17)
N3—C3—C4—C5	−0.49 (15)	N4—N3—C6—C7	91.53 (15)
N2—C3—C4—C5	179.30 (13)	N3—C6—C7—O3	−168.31 (14)
N3—C3—C4—C1	−179.86 (11)	N3—C6—C7—O2	13.70 (18)
N2—C3—C4—C1	−0.1 (2)		

### Hydrogen-bond geometry (Å, º)

*Cg*1 is the centroid of the C3/C4/C5/N4/N3 ring.

**Table:**

*D*—H···*A*	*D*—H	H···*A*	*D*···*A*	*D*—H···*A*
N1—H1···O1^i^	0.91	1.90	2.8094 (16)	175
O2—H2*A*···N2^ii^	0.87	1.85	2.7120 (14)	173
C5—H5···O1^iii^	0.93	2.33	3.2305 (18)	164
C6—H6*A*···O3^iv^	0.97	2.36	3.2436 (18)	152
C6—H6*B*···*Cg*1^v^	0.97	2.98	3.7387 (15)	136

Symmetry codes: (i) −*x*+1, −*y*+1, −*z*+1; (ii) −*x*+1/2, *y*+1/2, −*z*+1/2; (iii) −*x*+1/2, −*y*+3/2, −*z*+1; (iv) −*x*, *y*, −*z*+1/2; (v) *x*, *y*−1, *z*.
